# Specificity factors in cytoplasmic polyadenylation

**DOI:** 10.1002/wrna.1171

**Published:** 2013-06-14

**Authors:** Amanda Charlesworth, Hedda A Meijer, Cornelia H de Moor

**Affiliations:** 1 Department of Integrative Biology, University of Colorado DenverDenver, CO, USA; 2 MRC Toxicology Unit, University of LeicesterLeicester, UK; 3 School of Pharmacy, University of NottinghamNottingham, UK

## Abstract

Poly(A) tail elongation after export of an messenger RNA (mRNA) to the cytoplasm is called cytoplasmic polyadenylation. It was first discovered in oocytes and embryos, where it has roles in meiosis and development. In recent years, however, has been implicated in many other processes, including synaptic plasticity and mitosis. This review aims to introduce cytoplasmic polyadenylation with an emphasis on the factors and elements mediating this process for different mRNAs and in different animal species. We will discuss the RNA sequence elements mediating cytoplasmic polyadenylation in the 3′ untranslated regions of mRNAs, including the CPE, MBE, TCS, eCPE, and C-CPE. In addition to describing the role of general polyadenylation factors, we discuss the specific RNA binding protein families associated with cytoplasmic polyadenylation elements, including CPEB (CPEB1, CPEB2, CPEB3, and CPEB4), Pumilio (PUM2), Musashi (MSI1, MSI2), zygote arrest (ZAR2), ELAV like proteins (ELAVL1, HuR), poly(C) binding proteins (PCBP2, αCP2, hnRNP-E2), and Bicaudal C (BICC1). Some emerging themes in cytoplasmic polyadenylation will be highlighted. To facilitate understanding for those working in different organisms and fields, particularly those who are analyzing high throughput data, HUGO gene nomenclature for the human orthologs is used throughout. Where human orthologs have not been clearly identified, reference is made to protein families identified in man. © 2013 John Wiley & Sons, Ltd.

## THE POLY(A) TAIL, INITIAL SYNTHESIS, AND FUNCTION

We shall start with a brief overview of nuclear polyadenylation so that we can provide context for discussing cytoplasmic polyadenylation. For more detail, the reader is referred to other reviews that discuss nuclear polyadenylation specifically.^1^–^6^ The nuclear polyadenylation of eukaryotic messenger RNAs was discovered in the 1970s and soon was shown to be a nearly universal characteristic of this RNA species.^1^ In the following decades, polyadenylation was shown to follow cleavage of the newly transcribed messenger RNA (mRNA) and to be tightly coupled to transcription and to splicing of the 3′ intron.^1^–^3^ Cleavage of the nascent mRNA is now known to be required for normal transcription termination as well as for polyadenylation.^4^

**FIGURE 1:**
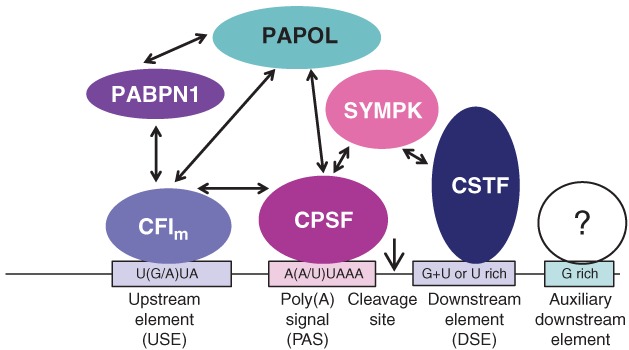
The nuclear cleavage and polyadenylation complex in vertebrates. The Cleavage and Polyadenylation Specificity Factor (CPSF) protein complex binds to the poly(A) signal PAS, it contains CPSF1-4 and the associated factors FIP1L and Symplekin (SYMPK).The CPSF3 subunit is the endonuclease acting at the cleavage site. The Cleavage Factor 1 complex (CFI_m_) recognizes the upstream element (USE), it is composed of NUDT21, CPSF6, and CPSF7. The CSTF complex recognizes the GU- or U-rich downstream element (DSE). CPSF, CSTF, SYMPK, and CFI_m_ interact at the protein level, stabilizing the RNA binding, thus promoting correct cleavage and polyadenylation site recognition and recruitment of the poly(A) polymerase (PAPOLA or PAPOLG). The nuclear poly(A) binding protein (PABPN1) interacts with CFI_m_ and PAPOLA and contributes to the efficiency of polyadenylation.^17^–^19^ Double arrows indicate interactions. The cleavage site is indicated by a bold single headed arrow. Some of these interactions have been inferred from work in yeast. For further details, see two recent reviews.^5^,^8^

The target selectivity of cleavage and polyadenylation in mammals is mediated by four sequence elements flanking the cleavage site, as depicted in Figure 1.^5^–^8^ Subunit 1 of the cleavage and polyadenylation specificity complex, CPSF1 (CPSF160), binds the well-known poly(A) signal (AAUAAA or AUU AAA), normally located 15–30 nucleotides upstream of the cleavage site. The CPSF complex also contains CPSF3 (CPSF73) the endonuclease responsible for cleavage,^9^ and FIP1L, the factor that recruits the poly(A) polymerase together with CPSF1.^10^,^11^ The cleavage stimulating factor (CSTF) complex binds to a GU or U rich sequence downstream of the cleavage site called the downstream element (DSE). The association of the CSTF and CPSF complexes with the RNA is thought to be the most important for the selection of the cleavage site, and the two complexes are connected by the 3′ processing scaffold protein Symplekin (SYMPK).^12^,^13^ The Cleavage Factor I (CFI_m_) is a complex of NUDT21 (CFI_m_25, CPSF5) and CPSF6 (CFI_m_68), or CPSF7 (CFI_m_59). NUDT21 binds upstream of the poly(A) signal to the upstream element (USE), which contains U(G/A)UA sequences that enhance recognition of the cleavage site.^5^,^14^ A fourth sequence element contributing to cleavage site recognition is a G rich sequence downstream of the DSE, for which the binding factor has not yet been determined.^5^ If the poly(A) signal diverges from AAUAAA or AUUAAA, the sequence of the other three sequences become more important for correct cleavage. Alternative poly(A) site selection has recently been shown to be regulated for many mRNAs and is likely to play an important role in post-transcriptional gene regulation by generating mRNAs with different 3′ untranslated regions (UTRs).^15^,^16^

After cleavage, the poly(A) tail is added by a nuclear poly(A) polymerase. The canonical enzyme that is thought to mediate this in most cells and for most mRNAs is poly(A) polymerase α (PAPOLA), which was originally isolated from calf thymus.^20^ It is required for both the polyadenylation and the cleavage reaction. However, another canonical mammalian poly(A) polymerase γ (PAPOLG) has been found in the nuclei of many tumor cells and cell lines^21^ and was the only poly(A) polymerase detected in a proteomic study of polyadenylation complexes.^22^ In addition, the noncanonical poly(A) polymerase TUT1 (Star-PAP, PAPD2) has been shown to mediate the nuclear polyadenylation of specific mRNAs.^23^–^28^

During cleavage and polyadenylation, a number of proteins are deposited on the mRNA which appear to limit the poly(A) tail size to 200–250 nt and play a role in mRNA export. These include nucleophosmin (NPM1), the nuclear poly(A) binding proteins PABPN1 and NAB2, as well as export factors.^29^–^32^ The removal of the cleavage and polyadenylation complex from the mRNA is also part of the export process.^33^

**FIGURE 2:**
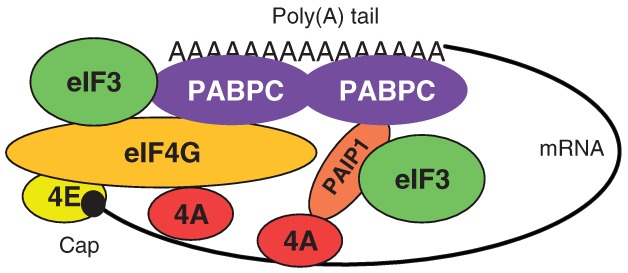
The closed loop complex enhances translation by increasing eIF4A and eIF3 recruitment. Cytoplasmic poly(A) binding proteins (e.g., PABPC1 or PABPC1L) stabilize the cap binding translation initiation complex consisting of eIF4E (4E), eIF4G, and eIF4A (4A), forming the closed loop complex. In addition, PABPC proteins can bind poly(A) binding protein interacting protein 1 (PAIP1), which, like eIF4G can bind eIF4A and the ribosome recruiting complex eIF3. This leads to enhanced translation mediated by the poly(A) tail. For references, see text.

In the cytoplasm, the poly(A) tail enhances translational efficiency and protects the mRNA from degradation.^34^,^35^ These functions are mediated by the cytoplasmic poly(A) binding proteins (PABPs), the most well characterized of which is PABPC1 (PABP1 or PAB1). Cytoplasmic PABPs first bind to the poly(A) tail in the nucleus and are exported with the newly made mRNA, where additional PABP molecules may bind.^36^–^38^ During translation, PABPs mediate the formation of the ‘closed loop complex’ (Figure 2) in which interactions between PABP and eIF4G, a subunit of the cap binding translation initiation complex, connect the ends of the mRNA. The cap binding complex recruits the ribosome through interactions with eIF3 and contains the eIF4A helicase, which unwinds the 5′ untranslated region of the mRNA. In addition, PABP can recruit further eIF4A and eIF3 through its interaction with PAIP1.^39^–^41^ In plants, a further PABPC1 interaction with the eIF4A binding factor eIF4B has been established^42^,^43^ and a similar complex may also exist in human cells.^44^ Thus, the poly(A) tail stimulates translation by remodeling of the mRNP (through eIF4A) and loading of ribosomes (through eIF3), all mediated by interactions with PABPCs.

The poly(A) tail also protects the mRNA from degradation, a process in which PABPs play a complex role, by both preventing and stimulating removal of the poly(A) tail, depending on the presence of different deadenylation complexes.^34^ Most mRNA degradation in eukaryotes is thought to occur by the deadenylation-dependent decapping pathway, in which the poly(A) tail must be shortened to 10–15 nt before the mRNA is decapped and degraded by exonucleases.^45^–^47^ The sequences regulating mRNA stability are usually found in the 3′ UTR of the mRNA, although elements in the 5′ UTR and the coding region can also participate. Deadenylation, the removal of the poly(A) tail by specific 3′ exonucleases, is a frequently regulated step in the determination of mRNA stability. Destabilizing 3′ UTR elements include AU and GU rich destabilizing elements that recruit proteins binding to deadenylating enzymes.^45^,^48^,^49^ However, mRNAs with short poly(A) tails are not rare in tissue culture cells^50^ and many 3′ UTRs can bind ELAVL1 (HuR) through U rich elements, which prevents the degradation of mRNAs with short tails.^51^–^53^ The poly(A) tail of an mRNA is therefore involved in its function throughout its lifetime, functioning in mRNA export, translation, and stability, and is the target of extensive regulation.

## THE ROLE OF CYTOPLASMIC POLYADENYLATION IN THE REGULATION OF GENE EXPRESSION

Cytoplasmic polyadenylation is the elongation of the poly(A) tail of an mRNA after it has been exported into the cytoplasm. Typically, this process increases protein expression from specific mRNAs by the translational activation of stored mRNAs with short poly(A) tails.^54^,^55^ In general mRNAs are thought to be synthesized with long poly(A) tails and the default state is for an mRNA to be translated upon exit from the nucleus. Therefore, regulation by cytoplasmic polyadenylation requires that the mRNA recruits factors that deadenylate and translationally repress the newly exported mRNA, as well as respond to a specific signal to induce expression by cytoplasmic polyadenylation

So far, the defined repressive elements are all in the 3′ untranslated regions of the mRNA and the same elements that convey activation by cytoplasmic polyadenylation sometimes can also mediate translational repression and mRNA deadenylation.^56^,^57^ In addition, these mRNAs often contain separate regions that recruit other deadenylation and translational repression factors.^58^–^64^ Especially widespread in early development are mRNAs with GU-rich elements, which bind the CELF family of deadenylation factors and mediate deadenylation in the *Xenopus* embryo, counteracting cytoplasmic polyadenylation activated in the oocyte.^49^,^65^–^68^ Although a shorter poly(A) tail generally reduces translational efficiency, the lack of a poly(A) tail alone is not sufficient for efficient inhibition of translation, as many translational repressors can reduce the expression from nonadenylated mRNAs significantly in cells.^56^,^69^ Consequently, in addition to recruiting deadenylating enzymes, mRNAs that are regulated by cytoplasmic polyadenylation also rely on other mechanisms to reduce translation efficiency, predominantly through preventing the association of the cap-binding translation initiation complex,^55^,^64^,^70^ although repression by interference with the elongation phase of protein synthesis has also been implicated recently.^71^ In addition, several deadenylation factors are able to repress translation directly, independently of their deadenylation activity.^70^,^72^ In microRNA-mediated translational repression, deadenylation is known to be secondary to efficient translational repression.^73^–^77^ It is not known if deadenylation follows or precedes translational repression in mRNAs repressed by other deadenylation factors.

The activation of translation by cytoplasmic polyadenylation is presumably mediated by the increased recruitment of poly(A) binding proteins to the elongated poly(A) tail and enhanced formation of the closed loop complex.^34^,^35^ However, a few studies indicate that active cytoplasmic polyadenylation or other remodeling of the mRNP is required for translational activation in addition to the presence of a poly(A) tail,^78^–^80^ as was reviewed previously in more detail.^70^ In theory, cytoplasmic polyadenylation should also be able to stabilize mRNAs that are dependent on deadenylation for their degradation.^45^–^47^ Interestingly, two such potential cases have been reported. In zebrafish embryos, the binding of a protein that appears to mediate cytoplasmic polyadenylation counteracts the destabilization of germline mRNAs by a microRNA.^81^ In addition, in the nematode *Caenorhabditis elegans*, mutation of the cytoplasmic polyadenylation machinery leads to a reduction in the levels of its target mRNAs.^82^

Regulation of translation by cytoplasmic polyadenylation allows nuclear export of mRNAs to be separated from the synthesis of the encoded proteins in time and space. Many of these mRNAs are indeed localized to specific positions in the cell, for instance Bicoid mRNA in early *Drosophila* embryos, CAMK2A mRNA in the dendrites of neurons and mRNAs encoding cell cycle regulators on the spindle of mitotic or meiotic cells.^83^–^86^ In several cases, the sequence elements and RNA binding proteins that mediate the control of poly(A) tail size also contribute to the intracellular localization of mRNAs. Cytoplasmic polyadenylation itself is therefore only one function of a family of RNP complexes that can also mediate translational repression, deadenylation, and mRNA localization.^54^,^55^,^70^,^87^ In the rest of this review we focus on the components of these complexes that determine which mRNAs are targeted by cytoplasmic polyadenylation, the cytoplasmic polyadenylation specificity factors (CyPSFs).

## CYTOPLASMIC POLYADENYLATION OF mRNAS IN GERM CELLS AND EMBRYOS

Cytoplasmic polyadenylation was first described in the 1970s in sea urchin embryos.^88^ Since then it has been observed in the oocytes and early embryos of many animal species including bivalves (*Spisula*), insects (*Drosophila*), amphibians (e.g., *Xenopus*), fish (e.g., zebrafish), and mammalians (e.g., mouse).^86^,^89^–^94^ In these developmental stages transcription is absent and posttranscriptional regulation is therefore particularly important and more readily detectable. The factors mediating cytoplasmic polyadenylation of mRNAs have predominantly been characterized in *Xenopus* oocytes and early embryos.^94^–^98^ The large size of *Xenopus* oocytes and embryos enables injection of radioactive RNA substrates, making it relatively easy to demonstrate that cytoplasmic polyadenylation occurs and to map the elements. Most of the mRNAs targeted by cytoplasmic polyadenylation in oocytes and early embryos are directly involved in meiosis and/or mitosis.^99^–^102^ In all cases in which this was examined, cytoplasmic polyadenylation in *Xenopus* requires the poly(A) signal that is also used for nuclear polyadenylation.^59^,^103^–^106^ In addition, specific sequence elements in the 3′ untranslated region are required. Five different sequence elements mediating cytoplasmic polyadenylation have been characterized in this system. Only one of these elements and its associated factors, the cytoplasmic polyadenylation element (CPE), has so far been studied in detail. Table 1 gives a summary of the evidence for the mRNA elements and specificity factors that may mediate cytoplasmic polyadenylation, as discussed further below.

**TABLE 1:** Summary of Potential Specificity Factors for Cytoplasmic Polyadenylation with Human Orthologs. For Further Details, See Text

Factor	Synonyms/Orthologs	Binding Site	Evidence for Cytoplasmic Polyadenylation
CPEB1	CPEB, Orb	**CPE** Strong: UUUUAU, UUUUAAU; Weak: UUUUACU,UUUUAAGU, UUUCAU^99^	CPE confirmed by polyadenylation in the absence of transcription and cytoplasmic injection of RNA in *Xenopus* and mouse oocytes, e.g.^107^–^110^ CPEB1 function shown by depletion from extracts and dominant mutants in *Xenopus*.^96^,^111^Good correlation in mammalian neurons and mitotic cells.^112^,^110^
CPEB4	Orb2	**CPE** Similar to CPEB1.^100^,^112^ Or higher affinity for U rich sequence with secondary structure.^113^	Injection into *Xenopus* oocytes late in maturation for CPE and knockdown for CPEB4. Correlation between element and protein function in mitotic cells^100^,^112^
MSI1	Musashi	**MBE** (G/A) U_1-3_ AGU^114^	Injection of RNA for the MBE and depletion as well as dominant negative mutants in *Xenopus* oocytes.^103^,^115^–^117^
ZAR2	Zygote Arrest 2	**TCS** (A/U)UU(A/G)UCU^59^	Injection of RNA demonstrated existence of TCS mediated polyadenylation in *Xenopus* oocytes.^58^,^59^,^63^ ZAR2 binds to the element but no other evidence for a function of this protein.^63^
PCBP2	αCP2, hnRNP-E2	**C-CPE** C-rich, e.g. CCCUC CCUCCUCCCC^105^	Injection of RNA into *Xenopus* embryos proved existence of the element and PCBP2 binds to this element, as well as to polyadenylation factors^105^
ELAVL1	HuR	**eCPE** U rich, U_12_^79^,^106^,^118^	eCPE function shown by RNA injection and by polyadenylation of endogenous mRNA in the absence of transcription in *Xenopus* embryos.^79^,^106^,^118^ ELAVL1 binds this element.^119^
PUM2	Pumilio, FBF1, FBF2	**PBE** UGUAU(A/U)UAU 120	In *Xenopus*, PUM2-CPEB1 interactions contribute to CPE mediated cytoplasmic polyadenylation in oocytes.^99^,^121^ In *C. elegans*, upregulates translation in association with PAPD4^122^
BICC1	Bicaudal C, GLD-3	Unknown	Interacts physically and functionally with PAPD4 and PAPD5 to activate target mRNAs in *C. elegans*.^123^,^124^
DAZL	Deleted in Azoospermia-like	GUU triplet^125^	No cytoplasmic polyadenylation observed in *Xenopus* oocytes, despite clear role in translational activation.^126^,^127^ In zebrafish embryos, DAZL binding negates microRNA mediated mRNA destabilization, and increases poly(A) tail size.^81^ Interacts with Pumilio.^128^
RBFOX2	RBM9, FOX-2	UGCAUG and others characterized in splicing substrates^129^,^130^	Binds to PAPD4 in the cytoplasm of *Xenopus* oocytes.^131^ No evidence that binding site confers cytoplasmic polyadenylation.
FMRP	Fragile X Mental Retardation Protein	Large number of widely different elements proposed e.g.^132^–^135^	Found to interact and co-localize with PAPD4 in *Drosophila* extracts and neurons.^136^ Functional evidence supports a role in translational repression rather than activation in oocytes.^137^

### The Cytoplasmic Poly(A) Polymerases in Oocytes and Embryos

Cytoplasmic polyadenylation must be mediated by poly(A) polymerases that are presumably recruited to specific mRNAs by CyPSFs and activated at the appropriate time. The best characterized cytoplasmic poly(A) polymerase, PAPD4 (Gld-2) was first discovered in *C. elegans* and its enzymatic activity was confirmed in mammalian orthologs.^91^,^138^ This enzyme is now thought to be the main polymerase associated with the CyPSF cytoplasmic polyadenylation element binding protein (CPEB) in full grown *Xenopus* oocytes,^95^ while in *Drosophila* oogenesis the CPEB homolog Orb first mediates cytoplasmic polyadenylation with the classical nuclear poly(A) polymerase (PAPOLA) before switching to a PAPD4 ortholog.^139^ *In vitro* studies indicated that *Xenopus* CPEB can also recruit PAPOLA to mediate polyadenylation and a cytoplasmic variant has been characterized, so it is possible that this poly(A) polymerase plays a role in cytoplasmic polyadenylation in vertebrate oocytes as well.^140^,^141^ However, a different study has reported that such truncated PAPOLA variants lack polyadenylation activity *in vitro*, so more evidence for a cytoplasmic role for this enzyme in oocytes is required.^142^ Although early studies indicated that PAPD4 is also the poly(A) polymerase involved in CPE mediated polyadenylation in mouse oocytes,^143^ knockout mice do not have changes in cytoplasmic polyadenylation in their oocytes.^144^ However, an ortholog of a third conserved poly(A) polymerase, PAPD5 (Gld-4) can compensate for PAPD4 (Gld-2) loss in oogenesis in *C. elegans*, and this protein can mediate CPEB1 dependent cytoplasmic polyadenylation in human fibroblasts, so perhaps such a compensation functions in mouse oocytes as well.^123^,^145^ A testis specific cytoplasmic poly(A) polymerase with high homology to PAPOLA, PAPOLB (TPAP), is required for sperm development in mouse, but it is unknown which CyPSF this enzyme is partnered with.^146^–^150^ This data therefore indicate that four poly(A) polymerases could participate in cytoplasmic polyadenylation in germ cell development in animals, and further research is required to determine which polymerases are involved in cytoplasmic polyadenylation in each stage of vertebrate oogenesis and development.

### The CPE and Its Binding Proteins

The CPE is a sequence that was originally characterized as conferring cytoplasmic polyadenylation during *Xenopus* oocyte maturation.^107^ Its first specific binding factor, now called CPEB1, was isolated from oocyte extract and shown to convey cytoplasmic polyadenylation.^96^ The four CPEB proteins conserved in vertebrates contain two RNA recognition motifs (RRMs), followed by a zinc finger domain located in the C terminus of the proteins.^151^,^152^ For CPEB1, all three of these domains are required for RNA binding.^153^

**FIGURE 3:**
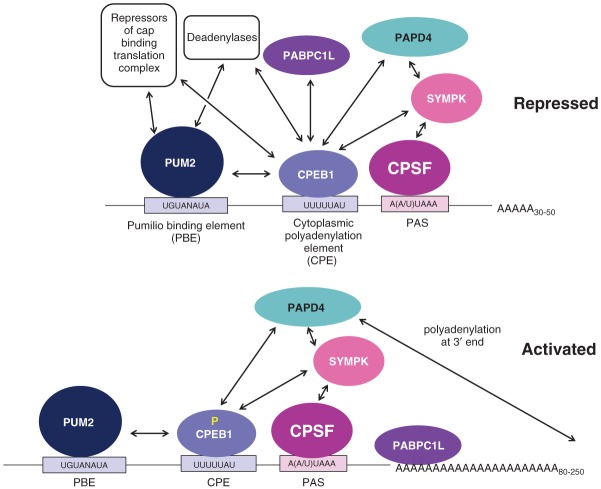
Cytoplasmic polyadenylation during *Xenopus* oocyte maturation. The cytoplasmic polyadenylation element (CPE) recruits its binding protein CPEB1.^96^ This binding can be stabilized, especially on noncanonical CPEs, by the recruitment of Pumilio (PUM2) to the Pumilio binding element (PBE) and protein–protein interactions between CPEB1 and PUM2.^99^,^121^ CPEB1 in turn interacts with the cytoplasmic CPSF complex, consisting of CPSF1,2 and 4 and the associated factor SYMPK (the endonuclease CPSF3 is absent and FIP1L has not been tested).^162^ CPSF1 recognizes the poly(A) signal (PAS). Before oocyte maturation translation is **Repressed**: CPEB1 and PUM2 mediate mRNA deadenylation and translational repression by recruiting deadenylases (CPEB1 recruits PARN,^163^ PUM2 can bind the CNOT complex^164^) and disrupting closed loop complex formation (PUM2 by direct cap interaction,^64^ CPEB1 by recruiting a choice of eIF4E binding or cap binding proteins^70^). In addition, CPEB1 binds the embryonic poly(A) binding protein PABPCL1.^165^ The poly(A) polymerase PAPD4 is secured in the complex by interactions with CPEB1 and CPSF, but its activity is repressed and/or masked by active deadenylation. Upon the stimulation of meiotic maturation, translation is **Activated**: CPEB1 is phosphorylated (yellow P).^166^ Deadenylases and translational repression complexes are ejected from the complex and/or inactivated,^163^,^167^ PAPD4 mediates elongation of the poly(A) tail^95^ and PABPC1L dissociates from CPEB1 and transferred to the poly(A) tail, where it can stimulate the formation of the closed loop complex and activate translation.^168^,^169^ More details and references can be found in the text.

CPEB1 and CPEB4 are required for normal *Xenopus* oocyte maturation and CPEB1 has been implicated in regulating the cell cycle in early embryos.^85^,^100^,^154^,^155^ CPEB1 knockout mice also have defects in oocyte and sperm development.^156^ In addition, CPEB homologs in *Drosophila* and *Caenorhabditis* have been shown to mediate translational activation and play a role in germ cell development and early embryogenesis, indicating that these functions are evolutionary conserved.^139^,^156^–^161^ Figure 3 summarizes the regulation of translation by CPEB1 as elucidated in *Xenopus* oocytes and discussed in detail below.

The CPE is a U-rich element containing a stretch of at least four U residues with a canonical CPE being UUUUAU or UUUUAAU, which binds CPEB1.^96^,^104^,^108^ All the variants that were used in a bioinformatic screen and correlate well with cytoplasmic polyadenylation in *Xenopus* oocytes can be found in Table 1.^99^ The RNA binding domains of mouse CPEB3 and CPEB4 have been reported to select a distinct structured recognition element containing a U rich bulge.^113^ However, *Xenopus* CPEB4 appears to target the same mRNAs as CPEB1 in oocytes and mediates cytoplasmic polyadenylation of the same mRNAs, although at a later stage of meiosis.^100^ Mammalian CPEB2 binds to sequences containing CPEs and binding can be competed with poly(U), indicating its binding site is also U-rich.^170^,^171^ So far, no structures of the RNA binding domains of CPEB family proteins in complex with RNA have been published, so the relationship between the structure of the protein and the RNA elements it binds remains unclear.

In addition to the CPE, the poly(A) signal that confers nuclear cleavage and polyadenylation (AAUAAA or AUUAAA) is required for CPE-mediated cytoplasmic polyadenylation in *Xenopus* oocytes.^104^ The protein complex recognizing the poly(A) signal is thought to be a form of CPSF, with the 160 kDa subunit (CPSF1) recognizing the RNA specifically. The 100 and 30 kDa subunits of CPSF (CPSF2 and CPSF4) are also present in the cytoplasm of *Xenopus* oocytes, while the 73 kDa endonuclease subunit (CPSF3) is absent.^172^ Because the poly(A) signal is present in nearly all native mRNAs, mRNA specific regulation must be mediated by binding sites for CyPSFs such as the CPE, but the regulation of cytoplasmic polyadenylation could in principle be influenced by factors associated with the poly(A) signal.

### Interaction Between the Complexes Associated with the CPE and the Poly(A) Signal

The spatial arrangement of CPEs in the 3′ untranslated region (3′ UTR) of an mRNA is important for their function.^99^ Two CPEs in close proximity (less than 50 nucleotides apart), mediate translational repression in immature oocytes regardless of their position in the 3′ UTR. In order to mediate robust cytoplasmic polyadenylation, a CPE normally has to be within 100 nucleotides of the poly(A) signal and ideally within 25 nucleotides. Cytoplasmic polyadenylation mediated by weak CPEs benefits from the presence of a second CPE in close proximity and/or the presence of the RNA recognition element UGUANUAU.^99^ This represents a binding site for the Pumilio (PUM2) RNA binding protein, which has a conserved interactions with CPEB proteins through protein–protein contacts and binds in their proximity on many mRNAs.^173^,^121^,^120^ Pumilio is thought to stabilize the binding of CPEB in this context, although it is also a deadenylation factor, a translational repressor and a putative CyPSF in its own right (discussed below).^64^,^174^,^175^,^122^,^164^ CPEB, in turn, associates with the CPSF complex bound to the poly(A) signal.^172^,^166^ CPEB also recruits the scaffold protein SYMPK, which itself can interact with CPSF.^95^,^176^ The cytoplasmic poly(A) polymerase PAPD4 is recruited by this complex, although the direct interactions are not yet clear. Cytoplasmic polyadenylation sequence recognition in mRNAs therefore has a multipartite character, with multiple elements recruiting proteins which also interact at the protein level. This is reminiscent of the determination of specificity in nuclear polyadenylation as depicted in Figures 1 and 3.

### Regulation of CPEB-Mediated Translational Activation in Oocytes

Activation of CPEB1-mediated cytoplasmic polyadenylation in *Xenopus* oocytes is through phosphorylation in its N terminal domains.^166^,^111^ This phosphorylation can be mediated by the Aurora kinase A (AURKA) and this leads to increased binding of CPSF and expulsion of the deadenylase PARN from the complex, shifting the balance to poly(A) tail elongation.^166^,^163^ The activation of CPEB1 mediated cytoplasmic polyadenylation by Aurora kinase phosphorylation is conserved in mice.^177^,^178^ However, several studies indicate that the earliest activation of CPEB1 in the *Xenopus* oocyte is mediated by a different kinase, with CDK1 activated by its noncyclin partner Speedy/Ringo (SPDY) being a strong candidate.^179^–^183^ For a more extensive discussion of the regulation of CPEB1 phosphorylation during *Xenopus* oocyte maturation, we refer to a previous review.^70^ The N-termini of the CPEB proteins are widely divergent,^151^,^152^ and the kinase activating other CPEB homologs is likely to be different. The *Drosophila* CPEB homolog Orb was recently shown to be activated through phosphorylation by casein kinase 2.^184^ In addition, the PAN GU kinase, which resembles the NimA (Never in mitosis A, NEK) family of kinases, regulates polyadenylation of cyclin A mRNA during meiosis in *Drosophila*,^185^ and is therefore a potential candidate for a regulator of cytoplasmic polyadenylation.

In addition to regulation by phosphorylation, the levels of CPEB proteins and their associated factors can also be regulated during oogenesis by increased synthesis or degradation. Increases are through translational activation, usually involving cytoplasmic polyadenylation. mRNAs regulated in this manner include CPEB4, Aurora kinase A (Eg2), and the cytoplasmic poly(A) polymerase PAPD4 (Gld-2) in *Xenopus* and Orb in *Drosophila*.^100^,^186^–^189^ CPEB1 is degraded during the completion of meiosis I through a mechanism involving phosporylation by a polo like kinase (PLK1) and the cyclin dependent kinase CDK1 (cdc2) and ubiquitination by the E3 ligase BTRC (SCF^β-TrCP^).^101^,^190^,^191^ This degradation has been proposed to be predominantly on nonRNA bound CPEB1 which is sequestered in the form of a dimer.^192^ SYMPK-bound CPEB1 levels, presumably representing RNA bound CPEB1 in polyadenylation competent complexes, remain unchanged during oogenesis.^95^

The timing of translational activation of mRNAs during the oocyte maturation is critical for the correct completion of meiosis. The Mos kinase is an early player in this signal transduction cascade^193^ and its synthesis is regulated by early cytoplasmic polyadenylation, while activation of cyclin B1 mRNA plays a later role, even though both these mRNAs contain CPEs and bind CPEB1.^80^,^84^,^108^,^194^,^195^ CPEs that do not overlap with the poly(A) signal have been reported to mediate earlier cytoplasmic polyadenylation. In contrast, the presence of a CPE overlapping with the poly(A) signal correlates with activation of cytoplasmic polyadenylation late in oocyte maturation, after the breakdown of the nuclear envelope in meioisis I.^99^,^195^ The reduction in CPEB1 levels during late oocyte maturation is thought to lead to the availability of the poly(A) signal in these mRNAs. The addition of AU-rich elements that bind deadenylation factors can also affect the timing of cytoplasmic polyadenylation.^196^ CPEB1-mediated polyadenylation is thought to be required for mouse oocyte maturation as well, and CPEB1 knockout mice fail to translate specific mRNAs very early in oogenesis, long before the full grown oocyte stages usually studied in *Xenopus*.^177^,^197^,^109^,^198^

### Other CPEB-Associated Factors that Influence Cytoplasmic Polyadenylation

A number of other proteins have been found associated with CPEB1 and are implicated in cytoplasmic polyadenylation in *Xenopus* oocytes. The poly(A) binding proteins PABPC1 (PAB) and PABPC1L (ePAB) both can bind to CPEB1. Embryonic poly(A) binding protein (PABPC1L) is the predominant poly(A) binding protein in vertebrate oocytes and embryos^199^,^200^ and is required for oogenesis in mice and oocyte maturation in *Xenopus*.^165^,^201^ PABPC1L phosphorylation is required for cytoplasmic polyadenylation in *Xenopus* oocytes and the protein transfers from CPEB1 to the poly(A) tail during cytoplasmic polyadenylation, protecting it from deadenylation.^165^,^168^

In addition, CPEB1 is bound by the GTP exchange factor xGEF, a member of the family of Rho guanine nucleotide exchange factors closely related to human ARHGEF39 (C9orf100).^202^,^203^ Early in oocyte maturation, xGEF recruits a kinase complex consisting of MAP kinase(ERK2) and a complex of CDK1 and its activator Speedy (SPDY, RINGO) to CPEB1.^179^,^180^,^204^ SPDY is required for the induction of cytoplasmic polyadenylation and is itself regulated by translational control.^205^,^126^ xGEF and SPDY are therefore likely to be involved in the activation of CPEB1 mediated polyadenylation during early oocyte maturation.

### Other mRNA Specific Cytoplasmic Polyadenylation in Vertebrate Oocytes

CPEB-mediated cytoplasmic polyadenylation is by far the best studied case, but there is very good evidence that it is not the only specificity factor that can confer cytoplasmic polyadenylation to mRNAs. Another well-established RNA binding protein that mediates cytoplasmic polyadenylation is Musashi (MSI1, MSI2).^206^ The MSI-binding element (MBE) (formerly the polyadenylation response element, PRE) was discovered when the role of the CPE was being investigated in a larger section of the Mos 3′ UTR than had been used previously.^107^,^115^ This new element conferred early cytoplasmic polyadenylation to the Mos 3′ UTR in oocytes that was maximal prior to meiosis I in the absence of CPEs and CPEB activity. Indeed, the TATA-BP2 mRNA is polyadenylated during oocyte maturation but does not have a recognizable CPE, only an MBE.^103^ MSI1 was identified as the *trans*-acting factor and like the MBE, shown to activate translation during oocyte maturation.^103^,^115^ A dominant negative MSI1 mutant and knockdown of MSI1 (and the partially compensating MSI2) using antisense oligodeoxynucleotides, prevented the polyadenylation of endogenous Mos mRNA and blocked maturation.^103^,^116^ This indicates that in addition to CPEB, MSI also plays an important role in cytoplasmic polyadenylation during oocyte maturation. Moreover, MSI1 is activated by phosphorylation that is dependent on Ringo/Speedy (SPDY) synthesis, and this phosphorylation is co-incident with Mos polyadenylation.^116^ Nonphosphorylatable MSI1 did not rescue oocyte maturation after removal of endogenous MSI, further strengthening the importance of MSI1 in oocyte maturation. The role of MSI1 in germ cell development and meiotic progression has also been described in *Drosophila* and mouse.^207^ Like the CPE, the MBE requires a poly(A) signal for cytoplasmic polyadenylation, but so far no interactions between MSI proteins and the polyadenylation machinery have been described.

The translation control sequence (TCS) is a third *cis*-element that mediates cytoplasmic polyadenylation during *Xenopus* oocyte maturation. The TCS was discovered when the role of CPEs was being investigated in the WEE1 3′ UTR.^58^,^59^ In the absence of CPEs, in both reporter constructs and the endogenous Pcm-1 mRNA, the TCS mediates early cytoplasmic polyadenylation, at least two hours prior to meiosis I. Like the CPE and the MBE, polyadenylation by the TCS requires the poly(A) signal. The TCS also confers translational repression in immature oocytes and translational activation during oocyte maturation. Recently, a *trans*-acting factor for the TCS has been found. Zygote arrest 2 (ZAR2) binds to the TCS and, like the TCS, represses translation in immature oocytes when tethered to a reporter RNA.^63^ This repression is relieved during oocyte maturation. ZAR2 and its homolog ZAR1 have been implicated in the early cleavage phase of mouse embryogenesis,^208^,^209^ supporting a function for these proteins in the regulation of early developmental processes. The role of ZAR2 in the regulation of polyadenylation has yet to be determined.

The single RRM protein DAZL has been shown to be critical for male and female germ cell differentiation, oocyte maturation and the oocyte-zygotic transition in several vertebrate species.^102^,^210^,^211^ In most cases, DAZL acts by inducing translational activation of its target mRNAs,^210^ but its binding site, a GUU triplet,^125^ has not been reported to convey cytoplasmic polyadenylation in *Xenopus* oocytes.^126^,^127^ In zebrafish embryos, DAZL binding regulates the stability of its target mRNAs, presumably to restrict their expression to the future germ cells, as this protein is normally germ cell specific.^81^,^212^–^214^ In one such study, DAZL was reported to overcome miR-430 mediated destabilization by the 3′ UTR of its own mRNA. This was accompanied by cytoplasmic polyadenylation of the injected mRNA.^81^ In contrast, in mouse oocytes, the translational activation and cytoplasmic polyadenylation of the Dazl mRNA was found to be mediated by CPEB1 and require a poly(A) signal.^102^ In *Xenopus* oocytes DAZL mediated translational activation appears not to involve cytoplasmic polyadenylation, but it requires an interaction between DAZL and poly(A) binding proteins.^126^,^127^ This interaction is highly conserved throughout the DAZL protein family and across species and cross-rescue experiments indicate that the mechanism of action is also conserved.^210^,^127^ However, DAZL can form a complex with the human Pumilio protein PUM2, so some DAZL functions may be mediated by Pumilo.^128^ More evidence is therefore required before DAZL protein can be truly considered a CyPSF. In addition, one publication indicates that another single RRM protein, RBFOX2 (Rbm9, Fox2), associates with PAPD4 in *Xenopus* oocytes and therefore also is a candidate CyPSF in this cell type.^131^

### Cytoplasmic Polyadenylation Mediated by Other Elements in *Xenopus* Embryos

Two more CPEs have been identified by deletion mapping of injected radiolabelled RNAs in *Xenopus*. Both mediate polyadenylation in early embryogenesis and not during oocyte maturation, which explains why they are less studied: good quality embryos are harder to obtain and inject than oocytes. First discovered, at about the same time as the CPE, was a stretch of 12 U residues that was called the eCPE.^106^,^215^,^94^ Like the CPE, it requires a poly(A) signal to function and the spacing between the eCPE and the poly(A) signal regulates the timing of polyadenylation.^79^,^106^,^118^ The eCPE can bind ELAVL1 (ElrA, HuR), but whether this is the genuine CyPSF is still uncertain.^119^,^216^

The second embryonic CPE in *Xenopus* was found to be a stretch of cytidine residues and called the C-CPE.^105^,^217^ Cytoplasmic polyadenylation by the C-CPE also requires a poly(A) signal and the element is bound by PCBP2 (αCP2, hnRNP-E2), which is exclusively cytoplasmic in *Xenopus* oocytes.^218^ PCBP2 immunoprecipitates with SYMPK, CPSF2, PAPD4, CPEB1, and PABPL1, but not with PARN, in embryos, and oocytes. This indicates that it is indeed part of a cytoplasmic polyadenylation complex that is pre-assembled in an inactive form in the oocyte. As was predicted by the PCBP2 and CPEB1 association, the C-CPE and the CPE cooperate to increase polyadenylation during early embryogenesis.^105^

### Other CPEs and Factors Found in Invertebrates

PAPD4 was originally discovered as germ-line deficient 2 (GLD-2) in *C. elegans*^91^ and a number of PAPD4-interacting RNA binding proteins that activate the translation of target mRNAs in different stages of germ line development have been characterized.^82^,^91^,^122^,^219^ Although the activity of PAPD4 as a poly(A) polymerase cannot be doubted, it has been much more difficult in this system to prove that cytoplasmic polyadenylation of specific mRNAs actually occurs in the germ cells and to map the sequences required for cytoplasmic polyadenylation on these mRNAs. PAPD4 RNA binding partners in *C. elegans* germ cell development include the Pumilio homologs FBF-1 and FBF-2 (PUM1, PUM2),^122^ RNP-8, a single RRM protein with no clear human homologs,^82^ and GLD-3, a KH domain protein with similarity to the *Drosophila* deadenylation factor Bicaudal C (BICC1).^220^–^223^ In addition to PAPD4, PAPD5 (GLD-4) has been identified as a cytoplasmic poly(A) polymerase in *C. elegans* and has been shown to promote germ line development with a complex of BICC1 and GLS-1, a protein with no clear human homologs.^123^,^224^ BICC1 is also implicated in male fertility in mice, indicating a conserved function in the germline.^225^

*Drosophila* Toll mRNA is regulated by cytoplasmic polyadenylation during early embryogenesis. Experiments using an *in vitro* polyadenylation system were used to map a 180 nucleotide region of the 3′ UTR that is required for this function.^226^ Competition with this RNA fragment indicated that the polyadenylation factors binding this region are distinct from those found in CPE containing mRNAs. Remarkably, the canonical nuclear poly(A) signal appears not to be required for the polyadenylation of Toll mRNA, indicating it employs a fundamentally different mechanism of cytoplasmic polyadenylation.^55^ The transacting factors mediating Toll mRNA cytoplasmic polyadenylation have not yet been characterized. Also in *Drosophila*, interactions of PAPD4 and CPEB (Orb) with fragile X mental retardation protein (FMRP) have been reported in neurons and oocytes, but its function appears to be opposite to that of CPEB.^136^,^137^

## CYTOPLASMIC POLYADENYLATION OF mRNAS IN SOMATIC CELLS

Elongation of the poly(A) tail has long been known to also take place in the cytoplasm of mammalian tissue culture cells, accounting for approximately 10% of all polyadenylation.^227^ However, cytoplasmic polyadenylation of specific mRNAs is much harder to study in somatic cells, as the synthesis of new mRNA also can contribute to changes in poly(A) tail size on specific mRNAs. Elongation of the poly(A) tail in a transcriptionally active cell can be due to reduced deadenylation of the poly(A) tail of newly made mRNAs as well as to cytoplasmic polyadenylation. Moreover, if only a relatively small portion of mRNAs in a cell undergoes poly(A) tail changes, for instance those localized under a particular set of synapses, the detection problem is further magnified. Determination of mRNA poly(A) tail sizes in most systems is dependent on techniques that are not very good at detecting rare mRNAs, such as variations on Northern blotting, or on PCR based methods that need to be very carefully controlled to avoid artifacts for a discussion see Ref 50. As a consequence, no new CPEs or specificity factors have so far been firmly identified in somatic cells. Some putative CyPSFs, such as DAZL and ZAR2, are very germ cell specific and unlikely to be of importance in somatic cells, with the possible exception of some tumor cells.^228^–^230^ However, a large body of work indicates that the CPEs and factors that function in germ cells and early embryos also are important in other cell types. It is not always clear, however, if cytoplasmic polyadenylation does indeed play a role in their function. Discussion of all cases where CyPSFs or elements have been implicated in mRNA regulation is outside the scope of this review. We have therefore selected some of the examples of regulation by CPEB, Pumilio, Musashi, PCBP and Bicaudal protein families, as these are confirmed or very likely CyPSFs in germ cells and early embryos.

### CPEB Family Proteins and PAPD4 in the Nervous System

In neuronal cells, the local regulation of translation in the dendrites is thought to play a role in the remodeling of synapses required for learning.^83^ CPEB1 protein and polyadenylation factors are present in the cell bodies and dendrites of many neurons and binds to mRNAs that are known to be partially dendritically localized. The CPEs in the 3′ UTRs of mRNAs such as calmodulin kinase II (CAMK2A) were found to be required for dendritic mRNA localization, as well as for translational stimulation and elongation of the poly(A) tail after synaptic stimulation.^231^–^238^ The activation of CPEB1 mediated cytoplasmic polyadenylation and translation in dendrites has been attributed to phosphorylation on the same site as in oocyte maturation, and the kinase is thought to be either AURKA or CAMK2A.^232^,^239^–^241^ As CAMK2A mRNA is also targeted by CPEB1, this is potentially another positive feedback loop, which has been proposed to contribute to the generation of a bistable switch.^242^ Another proposed mechanism of regulation of CPEB proteins in neurons is a prion-like conformation change in CPEB variants in *Aplysia* and *Drosophila*, although so far polyadenylation appears not to be involved.^243^–^246^

The polyadenylation inhibitor cordycepin has been used to show that polyadenylation is required for normal synaptic function.^113^,^233^ However, this type of experiment is no longer valid, because cordycepin is now known to inhibit protein synthesis through effects on the mTOR pathway in some cell types, as well as to have gene-specific effects on de novo mRNA synthesis.^247^,^248^ The most compelling early evidence that the poly(A) tail elongation observed in neuronal cells does not represent newly synthesized mRNAs comes from observation of poly(A) tail elongation *in vitro*, in synapse containing vesicles (synaptoneurosomes).^232^ Moreover, a recent study shows that a rapid increase in poly(A) (detected by *in situ* hybridization) does indeed occur in dendrites in response to NMDA receptor stimulation. The timing of this effect coincides with CPEB1 phosphorylation and PARN dissociation and it is abolished by knockdown of PAPD4 (Gld-2).^241^ Because of the speed of the response and the requirement for PAPD4, it is unlikely that this increase in poly(A) is due to the transport of newly made mRNA into the dendrites. This therefore probably represents a true case of cytoplasmic polyadenylation.

PAPD4 (Gld2) mutants in *Drosophila* have defects in long-term memory, which indicates that this enzyme is indeed important in synaptic plasticity.^136^ In addition, Orb2, the somatic CPEB in *Drosophila*, is required for asymmetric cell division during neuronal development, for normal locomotion and for memory formation.^249^,^250^ This suggests a major role for cytoplasmic polyadenylation in the *Drosophila* nervous system. CPEB1 has also been implicated in axon branching in hippocampal neurons, in cell migration of astrocytes and in motor skill development in rodents using expression of a CPEB1 dominant negative mutant.^251^–^253^ The Pumilio proteins, which can cooperate with CPEB1 in oocytes, have also been implicated in neuronal development and synaptic plasticity.^254^–^260^ However, *CPEB1* knockout mice have no major nervous system defects, although subtle effects on synaptic transmission and memory have been reported.^261^,^262^ This may be because other members of the CPEB family also are expressed in mammalian neurons.^113^,^263^ In particular, CPEB3 mediated translational control has been linked to changes in synapse function in rodents^264^,^265^ and a polymorphism in the *CPEB3* gene has been linked to memory in humans as well.^266^ An increase in poly(A) tail of a CPEB3 targeted mRNA has indeed been reported.^264^ In contrast to the regulation observed in oocytes, however, activation of translation is thought to be mediated by mono-ubiquitination or calpain mediated degradation.^264^,^265^ In addition, the activation of CPEB3 bound mRNAs does not require a poly(A) signal or CPSF.^113^ Similarly to the situation for CPEB1 in oocytes, CPEB3 is an efficient translational repressor^264^,^265^ and CPEB3 (and CPEB4) can associate with the TOB2 protein, which mediates deadenylation through recruitment of the CNOT deadenylation complex.^267^ The data so far are therefore inconclusive in determining if CPEB3 works as a deadenylation factor that is inactivated or as a CyPSF that is activated during synaptic stimulation in neurons.

### CPEB Family Proteins in the Regulation of Cell Proliferation

CPEB proteins have also been implicated in the regulation of mitosis, senescence and tumorigenesis, this has been recently reviewed in depth,^151^ with only one additional publication to note.^171^ In most cases where cytoplasmic polyadenylation has been implicated, clear effects of CPEB proteins on translational efficiency were detected. However, actual cytoplasmic polyadenylation in somatic cells other than neurons has not been definitively demonstrated. One approach has been to inject RNA into *Xenopus* oocytes to show that the 3′ UTR can mediate cytoplasmic polyadenylation.^170^,^231^ Unfortunately, this is no definitive guarantee that translational or poly(A) tail changes are caused by cytoplasmic polyadenylation in the tissue of interest. Another study shows a suggestive correlation between phosphorylation of CPEB1 and poly(A) tail elongation.^268^ Only two articles come close to demonstrating that CPEB mediated cytoplasmic polyadenylation genuinely plays a role, one in mitosis and one in senescence,^145^,^112^ these are discussed below.

In early embryos, cell division is atypical, with an absence of the G1 and G2 phases, so the finding that CPEB1 activates translation by cytoplasmic polyadenylation during mitosis in the embryonic cell cycle^85^,^269^,^270^ did not have immediate implications for the normal somatic cell cycle. However, most mRNAs regulated by cytoplasmic polyadenylation in oocytes encode proteins that are also involved in normal mitosis.^151^ In addition, mRNA transcription is known to be reduced in G2 and blocked in M phase in HeLa cells,^271^ similar to the situation in oocytes. In the first systematic screen for differentially polyadenylated mRNAs in mammalian cells, Novoa *et al*.^112^ compared the poly(A) tail sizes of mRNAs in HeLa cells in the S and G2/M phases. Total poly(A) RNA was isolated using oligo(dT) chromatography and RNA with short poly(A) tails was isolated by low stringency elution of poly(U) chromatography. Hundreds of mRNAs displayed cell cycle dependent changes in the ratios between the total and short poly(A) tail fractions, indicating that poly(A) tail regulation is widespread during the cell cycle. Knockdown of CPEB1 and CPEB4 caused cell cycle defects and affected the polyadenylation of some, but not all of these mRNAs during G2/M. These data indicate that both CPE dependent and other types of cytoplasmic polyadenylation are occurring during the G2/M phase of the cell cycle in HeLa cells.

In primary fibroblasts, knockdown or knockout of CPEB1 prevents senescence, a process that halts cell proliferation in normal cells after a finite number of cell divisions.^272^,^273^ The translational activation of the tumor suppressor TP53 (p53), a factor required for senescence, is dependent on CPEB1 and TP53 mRNA has a short poly(A) tail in human fibroblasts with reduced CPEB1.^272^ Surprisingly, the knockdown of the PAPD4 (Gld2) poly(A) polymerase increased TP53 translation, an effect that was attributed to the role of PAPD4 in the maturation of a microRNA that targets CPEB1 mRNA in these cells, leading to increased CPEB1 levels.^145^ Knockdown of the PAPD5 (Gld4) poly(A) polymerase did reduce TP53 protein expression and TP53 mRNA polyadenylation, and PAPD5 was found associated with CPEB1 and the TP53 mRNA.^272^ These data indicate that TP53 mRNA is regulated by polyadenylation mediated by CPEB1 and PAPD5 during the senescence of human fibroblasts. However, since both CPEB proteins and PAPD5 can also be found in the nucleus in somatic cells^274^–^277^ and transcription is ongoing in these experiments, an effect on nuclear polyadenylation cannot be excluded.

### Other Roles of CyPSFs in Somatic Cells

An exciting recent study demonstrated widespread regulation of poly(A) tail size during the circadian rhythm in the liver, some of which appears to be linked to transcriptional up regulation and some of which are not linked to transcriptional increases.^278^ The poly(A) tail sizes of some of the mRNAs in the last class are regulated by CPEB1 and CPEB2. Less well characterized and new polyadenylation regulatory elements are likely to be involved in the regulation of the other mRNAs identified in this study.

Musashi (MSI1 and MSI2) was initially characterized as a factor that promoted the self-renewal of neural stem cells by translationally repressing numb, an inhibitor of Notch signaling.^279^ More recently, MSI has been implicated as a marker for a variety of stem cells including mammary cells,^280^ normal and malignant gasterointestinal cells,^281^,^282^ endometrial stem cells,^283^ and in normal and malignant hematopoesis.^284^ The mechanism of translational repression by mammalian MSI in somatic cells is proposed to be the disruption of the closed loop translation initiation complex due to an interaction of MSI1 with PABP.^285^

The Pumilio family (PUM1 and PUM2) proteins are highly conserved in eukaryotes and well characterized as deadenylation factors and translational repressors in many cell types.^286^ In addition to their functions in neurons (see above), Pumilio proteins have been implicated in the regulation of MAPK signaling and cell proliferation.^287^–^289^ There is so far no evidence that these factors can mediate cytoplasmic polyadenylation in somatic cells, whether on their own or in conjunction with CPEB proteins.

The Bicaudal C homolog *BICC1* is linked to polycystic kidney disease in zebrafish, *Xenopus*, mouse and human,^290^–^292^ this is caused by defects in epithelial morphogenesis.^292^–^295^ CPEB1 deficiency also causes epithelial defects in the breast tissue of mice.^296^ These observations complement the long established localization of the polyadenylation factor SYMPK at tight junctions,^297^,^298^ and suggest a potential function for local translation mediated by cytoplasmic polyadenylation in the morphogenesis of epithelia. BICC1 appears to be a KH domain RNA binding protein, but a consensus binding element in RNA has so far not been identified.^222^,^299^ It has been reported to both to enhance and counteract microRNA mediated repression.^300^,^301^ No data are available on the effects of BICC1 on mRNA polyadenylation in somatic cells.

Both PCBP2 (αCP2) and ELAVL 1 (HuR) are best known as proteins that mediate mRNA stabilization in somatic cells,^302^,^303^ but there is so far no evidence that this role involves cytoplasmic polyadenylation. PCBP2 and its closely related family member, PCBP1 (hnRNP-E1), have been shown to mediate translational repression in haematopoietic cells.^304^,^305^ Strikingly, the C-CPE that can promote cytoplasmic polyadenylation in *Xenopus* embryos^105^ and mRNA stabilization in the cytoplasm of somatic cells also mediates gene-specific increased splicing, cleavage and polyadenylation of pre-mRNA in the nucleus.^306^ In addition, PCBP2 stimulated polyadenylation and was found to associate with three CPSF subunits and SYMPK in nuclear extracts. This observation opens the possibility that other cytoplasmic polyadenylation sequences and factors also can influence nuclear polyadenylation, and conversely, that proteins that bind upstream polyadenylation elements in the nucleus may also have a function in cytoplasmic polyadenylation. In this context it is remarkable that a subunit of the USE binding factor CFI_m_ (NUDT21) has been found together with PCBP1 in a vinculin bound cytoplasmic RNP complex during cell adhesion.^307^ Moreover, the NUDT21 recognition site is included in the Pumilio binding element, opening the possibility of competitive or subsequent binding.^120^,^308^ Interestingly, gene specific increases in splicing and nuclear 3′ mRNA processing have been shown to play a major role in the regulation of cyclin expression during meiosis in yeast,^309^ and regulation of nuclear polyadenylation by USEs may therefore be the ancestral mechanism that evolved into cytoplasmic polyadenylation in animal germ cells.

## CONCLUSIONS

Cytoplasmic polyadenylation was first characterized in *Xenopus* oocytes, and all well characterized examples of regulatory sequences are still from this system, with the best established specificity factors being CPEB1, CPEB4, MSI1, and PCBP2. However, a role for cytoplasmic polyadenylation is also emerging in the *C. elegans* germ line, as well as in neuronal plasticity and mitosis in somatic cells. While conclusive evidence of cytoplasmic polyadenylation still is dependent on showing that poly(A) tail elongation is independent of transcription, good circumstantial cases can be made where mRNA activation can be linked to the physical and functional association of an RNA binding protein in complex with one or more poly(A) polymerases, such as has been shown for BicaudalC and Pumilio in *C. elegans*. However, due caution must be exercised when assigning cytoplasmic mRNA associated functions to poly(A) polymerases, as most are present in the nucleus as well and these enzymes are also involved in the processing and degradation of noncoding RNAs.^274^,^310^–^312^ Increasingly, complexes of multiple RNA binding proteins are implicated in the mRNA specificity as well as the timing of cytoplasmic polyadenylation. The best characterized is the CPEB-Pumilio interaction, but now there are also the CPEB1-PCBP2 and Pumilio-DAZL interactions and many mRNAs that are regulated by cytoplasmic polyadenylation in oocytes have binding sites for multiple factors. This indicates that cooperation and/or antagonism of different poly(A) regulatory factors is common. Cytoplasmic polyadenylation now appears to be a widespread phenomenon, and is increasingly attracting the interest of researchers working in systems other than *Xenopus* and *C. elegans* oogenesis and early development. Therefore, we can expect new specificity factors to emerge in the near future.
